# Mediated Leader Effects: The Impact of Newspapers’ Portrayal of Party Leadership on Electoral Support

**DOI:** 10.1177/1940161217740696

**Published:** 2017-11-28

**Authors:** Loes Aaldering, Tom van der Meer, Wouter Van der Brug

**Affiliations:** 1 University of Vienna, Vienna, Austria; 2 University of Amsterdam, Amsterdam, The Netherlands

**Keywords:** media effects, election campaign, political leadership, Western Europe, voting behavior, content analysis, panel data

## Abstract

Conventional wisdom holds that party leaders matter in democratic elections. As very few voters have direct contact with party leaders, media are voters’ primary source of information about these leaders and, thus, the likely origin of leader effects on party support. Our study focuses on these supposed electoral effects of the media coverage of party leaders. We examine the positive and negative effects of specific leadership images in Dutch newspapers on vote intentions. To this end, we combine an extensive automated content analysis of leadership images in the media with a panel data set, the Dutch 1Vandaag Opinion Panel (1VOP), consisting of more than fifty thousand unique respondents and 110 waves of interviews conducted between September 2006 and September 2012. The results confirm that media coverage of party leaders’ character traits affects voters: Positive mediated leadership images increase support for the leader’s party, while negative images decrease this support. However, this influence is not unconditional: During campaign periods, positive leadership images have a stronger effect, while negative images no longer have an impact on subsequent vote intentions.

## Introduction

*Since the media focus so heavily on leaders’ personalities—what they are doing and saying, and where they are in the “race”—it is natural that voters, as consumers of the media, are also likely to focus heavily on party leaders when making their choice at the ballot box*. (Bittner 2011: 92)

In her book *Platform or Personality? The Role of Party Leaders in Elections*, Bittner (2011) argues that party leaders are important electoral forces in democratic elections. As the spokespersons of political parties, party leaders are the most visible political actors and most clearly convey a party’s message to voters. Based on an analysis of thirty-five election studies across seven countries, Bittner shows that voters’ assessment of (the personality of) party leaders strongly affects their party preferences.

While preferences for party leaders are difficult to distinguish from preferences for their parties, recent research shows that party leaders have an independent electoral effect. Research shows that party leaders reinforce pre-existing party preferences but also make voters defect from parties. Indeed, voters may change their party preferences and vote choice because they value the party leader of a competing party to a greater extent than they value the party leaders of their own party. This phenomenon we call *leader effects*^1^ (e.g., Aarts et al. 2013; Bittner 2011; Lobo and Curtice 2014; Mughan 2000).

The media are likely to play an important role in the way political leaders influence society (Bittner 2011). Most voters never meet party leaders in real life, and therefore, they form their judgments about them mainly on the basis of their representation in the media (e.g., Esser and Strömbäck 2014; Robinson 1976; Strömbäck 2008). Thus, media coverage of party leaders is a likely source of electoral leader effects. Our study focuses on the extent to which media coverage of party leaders affect support for their parties. We refer to the media coverage of party leaders in terms of their leadership traits as “mediated leadership images.”

Three distinct lines of research suggest that mediated leadership images affect electoral behavior. First, the literature on mediatization suggests that the media’s dominance over political parties in terms of determining the content of political news reports is increasing. The resulting media logic—characterized by a focus on conflict, horse race news, and the personalization of politics—places a particularly strong focus on party leadership (Altheide and Snow 1979; Mazzoleni 1987; McAllister 2007). Second, the literature on political leadership and electoral behavior suggests that voters’ perceptions of the character of party leaders affect their vote decisions. Positive evaluations increase the likelihood of voting for the leader’s party, and negative evaluations decrease this likelihood (Bean and Mughan 1989; Bittner 2011; Miller et al. 1986). Third, the literature on electoral volatility suggests that changes in vote intentions and decisions are caused by short-term fluctuations that are dynamic in nature. While most voters remain committed to a small and rather stable choice set of parties, their final choice within this choice set is likely determined by short-term concerns, such as images of leaders, party performance, and strategic incentives (e.g., Miller and Shanks 1996; Stokes et al. 1958; van der Meer et al. 2012).

Even though mediated leadership images are theoretically expected to have important electoral consequences (Bittner 2011; King 2002), direct tests of this effect remain largely lacking. Our study fills this void in the literature, by testing mediated leader effects on vote intentions. To study these effects, we combine two unique data sets in the Netherlands. The first is an extensive automated content analysis of Dutch newspapers’ coverage of leadership traits between 2006 and 2012, measuring positive and negative images of a leader’s political craftsmanship, vigorousness, integrity, communicative skills, and consistency (Aaldering and Vliegenthart 2016). The second is a panel data set, the Dutch *1Vandaag Opinion Panel* (1VOP), which consists of more than fifty thousand unique respondents and 110 waves of interviews covering vote intentions in the two governmental periods between September 2006 and September 2012. As we will discuss in more detail below, the Netherlands is a case in which mediated leader effects are not expected to be particularly strong, nor particularly weak. On the one hand, its institutional environment, combined with the fact that newspapers are relatively loosely connected to parties, are expected to depress mediated leader effects. On the other hand, the multiparty system, which offers many options for switching between ideologically similar parties, is expected to increase the impact of media coverage of party leaders on voting behavior.

The results of this study show that positive images of leadership in the media increase the leader’s party support, while negative images decrease this support. Our findings clearly highlight the significance of leadership images in the electoral process and show that the media not only affect what their users think about but also how they think about it. However, these effects vary across time. During campaign periods, the negative images of leadership in the media no longer undermine the likelihood to vote for that leader’s party.

Overall, the contribution of this research is twofold. First, it extends the causal chain from subjective perceptions of leaders on electoral behavior by including mediated images of leadership. Second, it contributes to the growing body of research on the direct effects of media content on public opinion, especially by showing the impact of the election campaign.

## Mediated Leader Effects and Character Traits

Classical models of voting behavior—the Columbia school, the Michigan school, and the Downsian rational choice model—pay little attention to the influence of leaders. However, this topic has been well studied in recent electoral research. Various studies show that favorable subjective evaluations of a party leader increase support for that leader’s party (e.g., Aarts et al., 2013; Bittner 2011; Lobo and Curtice 2014; Mughan 2000; but see also King 2002). The scope of these subjective leader effects remains contested and is argued to be contingent on a range of factors including political institutions (e.g., Curtice and Hunjan, 2013), the societal context (e.g., Gidengil 2013), and partisan de-alignment (e.g., Kriesi 2012).

The electoral effects of subjective leader evaluations are a necessary but insufficient condition to test leader effects. Feelings toward the party leader cannot be easily distinguished from feelings toward the party as a whole (Bittner 2011; Garzia 2012; Holmberg and Oscarsson, 2013; Johnston 2002; Page and Jones 1979). The evaluation of the party leader influences how voters rate parties, but party identification also influences perceptions of the party leader. When we study the extent to which media reports on party leaders affect voters’ (changing) vote preferences, we largely bypass this endogenous relationship. Surprisingly, little empirical research has been conducted on the way media portrayals of party leaders affect citizens’ voting behavior, although the scarce literature on mediated leader effects suggests that media coverage of party leaders indeed influences voters’ perception of party leaders and subsequently their vote decision (e.g., Bos et al. 2011; Eberl et al. 2017; Kleinnijenhuis et al. 2007; McCombs et al. 1997).

Why would mediated images of leadership matter? The classic agenda-setting hypothesis (McCombs and Shaw 1972) argues that the media influence who and what users think about when casting a ballot. Hopmann et al. (2010), for instance, show that a party’s visibility in media coverage increases the number of voters who are inclined to vote for that party. We can easily transpose this rationale to understand mediated leader effects. The prominence of party leaders in news coverage is likely to affect voters’ vote decision (Bos 2012; Kiousis and McCombs 2004), and indeed, for instance Mughan (2000) finds this positive relation. Moreover, the media transfer to the public not only object salience but also attribute salience; the way things are presented in the media affects voters perceptions (understood as framing, for example, De Vreese and Boomgaarden 2003; Lecheler and De Vreese 2012; Scheufele 2000; or second-level agenda setting, for example, McCombs et al. 1997).^2^ Thus, the media influence the public agenda by affecting what people think about and how they think about it. Based on this logic, we propose the following:

**Hypothesis 1:** *Party leader tone effect*: As the number of positive (negative) images of leadership in the media increases, the propensity that voters will switch to that leader’s party increases (decreases).

Most research on the effects of media content on electoral behavior focuses exclusively on campaign periods (e.g., Ansolabehere et al. 1991; Bos et al. 2011; Gerber et al. 2009; Kleinnijenhuis et al. 2007; Kleinnijenhuis and Ridder 1998; Vreese and Semetko 2004). The scarce number of studies that cover a longer time span either focus on media exposure rather than on the content of the messages (e.g., Bartels 1993; van der Meer et al. 2015) or rely on aggregate-level party support rather than individual voters’ changes (e.g., Boomgaarden and Vliegenthart 2007; van der Pas et al. 2013; Vliegenthart et al. 2012). This paper goes beyond this limitation and studies mediated leader effects on individual voters’ vote intentions in a six-year period, including three national election campaign periods and the periods of routine politics between these elections.

The distinction between campaign periods and routine periods is relevant in three respects. First, the link between the content of media coverage and public opinion might be particularly apparent during campaigns. In this period, both the media and the public pay more attention to politics and party leaders than they do in “routine” times, enhancing the possibility of the media to influence public opinion (Soroka et al. 2009). Second, the nature of electoral change likely differs during routine and campaign periods. Because voters require less-defined vote intentions—as their choice process does not need to be complete (Tillie 1995)—citizens are likely to respond to different triggers outside campaign periods. The lack of focus on upcoming elections allows for more retrospective, emotional, and idiosyncratic attitude changes during routine times, while short-term factors, such as party leaders, should have a larger impact on voters during campaign periods. Third, the news coverage becomes more personalized in the run-up to the elections, that is, the media’s attention paid to party leaders increases as the elections approach, as manifested in increased attention to leader debates, campaign events and internal party turmoil (Takens et al. 2015). Research shows that exposure to personalized news coverage increases the influence of perceptions of party leaders on the vote decision (ibid.), indicating that leader effects in general are stronger during campaign periods than during routine times. Overall, we expect the electoral impact of mediated leadership images to be larger during campaign periods, which leads to the following hypothesis:

**Hypothesis 2:** *Party leader campaign effect*: The effects of mediated leadership images on shifting vote intentions are stronger during campaign periods than during routine times.

Extant research based on prospect theory has shown the existence of a *negativity bias* (Kahneman and Tversky 1979; Tversky and Kahneman 1992): Responses to negative information tend to be stronger than responses to positive news. These asymmetrical reactions to positive and negative triggers are found in a range of fields, including economic information (Soroka 2006), campaign information (Lau and Pomper 2002), and perceptions of political parties and party leaders (e.g., Holbrook et al. 2001; Klein 1991; Lau 1982; but see also Aarts and Blais 2013; Wattenberg 1991). Following this argument, we expect the push factors in a leader’s image to be more important than the pull factors:

**Hypothesis 3:** *Party leader negativity bias*: The effect of negative mediated leadership images on shifting vote intentions is stronger than the effect of positive mediated leadership images.

Bittner (2011), among others, argues that to understand leader effects, we should study leaders’ character traits. Voters likely base their general judgment of party leaders on their judgments of these leaders’ wide range of characteristics (Greene 2001; Shabad and Andersen 1979; Ohr and Oscarsson 2013). In addition, evaluations on character traits can be measured more reliably than general leader perceptions (e.g., Schwarz 1998). But which character traits do voters desire in their political leaders? This study employs a typology of leadership character traits developed by Aaldering and Vliegenthart (2016), who begin by conducting an extensive literature review on leadership traits and find that other scholars distinguish two (e.g., Bittner 2011; Johnston 2002), three (e.g., Funk 1999), four (e.g., Kinder 1986; Miller et al. 1986), six (e.g., Bass 1981), and up to fourteen character dimensions (Simonton 1986). Aaldering and Vliegenthart’s (2016) conceptualization integrates existing typologies in an overarching framework, which consists of five empirically relevant leadership character traits.

First, *political skills* include the skills needed to perform well in the political arena, such as general competence, political intelligence, and strategic behavior. Second, strong and powerful leadership, (self-)confidence, and decisive behavior by party leaders are called *vigorousness*. Third, *integrity* refers to leaders’ honesty, (un)corruptness, and whether the leader is focused on his or her own needs or on the needs of the electorate. Fourth, *communicative skills* refers to both inspiring or visionary leadership and the mediagenic skills of the leader, including whether the leader comes across as friendly, clear, empathic, and charming. Finally, the stability of leaders’ visions and actions is labeled *consistency* and includes whether the leader behaves in a predictable manner.

Studying the electoral effects of a party leader’s mediated personality, the question arises if voters are influenced mainly by general media coverage or by the discussion of specific traits. Most scholars argue that specific traits are important, although the literature remains inconclusive in identifying the characteristics that matter most. Miller et al. (1986), for instance, find that performance-related character traits, such as competence and reliability, matter most to voters. Others additionally show strongest effects for competence (e.g., Bean and Mughan 1989; Lewis-Beck and Nadeau 2014). However, Johnston (2002) and Bittner (2011) find that party leaders’ integrity and empathy affect citizens strongest. Based on these conflicting results, we cannot formulate clear expectations on the most influential (set of) character trait(s). However, these findings demonstrate that voters’ vote choice is affected by perceptions of party leaders in terms of specific characteristics. Therefore, we expect to find electoral effects not only for the media’s general tone about a party leader’s personality but also for the media’s discussion of party leaders’ specific characteristics:

**Hypothesis 4:** *Party leadership trait effect*: As the number of positive (negative) images in the media in terms of a party leader’s (a) political craftsmanship, (b) vigorousness, (c) integrity, (d) communicative skills, and (e) consistency increases, the likelihood that voters will switch to the leader’s party increases (decreases).

## The Dutch Case

Most research into leader effects focused on the United States or the United Kingdom. There are reasons to expect leader effects in the Dutch context to be smaller than in both of these countries. For one, leader effects are usually stronger in presidential systems than in parliamentary systems (e.g., Bittner 2011; Curtice and Holmberg 2005; Holmberg and Oscarsson 2013). The difference in the scope of leader effects is often explained by the fact that people vote for a candidate (who receives a personal mandate for a fixed period in time) in the former and for a party (lacking a personal mandate and, when governing, depending on parliamentary support) in the latter (McAllister 1996; Wattenberg 1991). Furthermore, within parliamentary systems, the electoral impact of leaders is generally larger in majoritarian systems than in proportional systems (as in the Netherlands), particularly because the accountability of parties and party leaders is less straightforward in multiparty systems with coalition governments than in two-party systems (Curtice and Hunjan 2013).

Another reason not to expect particularly strong mediated leadership effects is that Dutch newspapers do not have strong partisan ties, nor very strong ideological leanings. To be sure, the largest Dutch newspaper *De Telegraaf* is seen as right-wing and its reporting of the right-wing Volkspartij voor Vrijheid en Democratie (﻿VVD) will tend to be favorable, while the newspaper *De Volkskrant* is seen as left-leaning and will be more critical of right-wing politicians. However, the type of strong partisan support that we see in the United States (e.g., Fox news) and the United Kingdom (e.g., the Sun) is of a very different magnitude (e.g., Brants and Van Praag 2006; Hallin and Mancini 2004). Given that Dutch newspapers can be expected to report in a relatively more neutral tone on leaders, the electoral effects of mediated leadership images can be expected to be moderate.

Despite these features that are expected to dampen the magnitude of leader effects in the Dutch context, other aspects likely induce such effects. Even though newspaper readership is declining in the Netherlands, like elsewhere, the proportion of citizens that read newspapers on a regularly basis is relatively high. In 2005, one year before the start of the research period under study, 55 percent of Dutch households received a copy of a paid daily newspaper. When free newspapers are also accounted for, about 71 percent of Dutch citizens read a newspaper on a daily basis (Bakker and Vasterman 2007: 146).

Another feature of the Netherlands that can be expected to induce leadership effect is the highly proportional electoral system of the Netherlands, which usually results in the ten or more parties obtaining seats in Parliament. Because of the availability of so many alternatives, many voters switch easily between two or more ideologically similar parties (van der Meer et al. 2012). In a two-party system, it is a major step for most voters to switch sides, while for a Dutch voter who normally votes for the social democrats and dislikes the current leader, it would be a much smaller step to change to another left-wing party.

In sum, the Netherlands is a case with features that dampen the influence of party leaders on the vote decision, as well as aspects that induce mediated leader effects. So, compared with other countries, we do not expect these effects to be particularly strong, nor exceptionally weak.

## Data and Method

To test the electoral effects of mediated leadership images, media data are combined with panel data in the so-called linkage-analysis (Scharkow and Bachl 2017), a research tradition that can be considered state of the art when testing the influence of news content of political behavior (Fazekas and Larsen 2016).

### Content Analysis

We gathered media images of party leaders via computer-assisted content analysis of all newspaper articles that were published in all major daily national newspapers in the Netherlands^3^ in the period between September 1, 2006, and September 12, 2012 (Election Day of the 2012 elections). The period of this study includes the campaign periods of three national parliamentary elections and two long routine periods between the elections. We covered the leaders of all political parties with at least one elected seat in Parliament,^4^ resulting in twenty-one party leaders^5^ representing eleven different parties. To analyze media reports, we applied the measurement instrument of images of political leadership based on the dictionary approach manually constructed by Aaldering and Vliegenthart (2016). The validity of the automated content analysis was examined and cross-validated with manually coded content analysis, along with other tests.^6^

The media data include, first, party leaders’ visibility in newspapers, that is, the average number of newspaper articles that refer to the party leader per day. More importantly, we study mediated images of political skills, vigorousness, integrity, communicative skills, and consistency. For each of these five leadership traits, two dictionaries are manually constructed: one that taps into positive images of this character trait and one tapping into negative ones. Thus, ten specific leadership images for each party leader are studied in total. We chose not to include a net-tone measurement (positive minus negative) for each trait, as we assume asymmetrical reactions to positive and negative media coverage.

The dictionary search was conducted on newspaper articles that combine a reference to a party leader with a reference to a leadership image (with a maximal distance of five words). To this end, the dictionaries include words and word combinations that measure the appearance of these images in newspaper articles, additionally including the negation of the absence of that image; for instance, we included newspaper articles that refer to a party leader in combination with phrases that capture a positive image of consistency (e.g., “predictable” and “keeps its promises”) and with phrases that capture the negation of a negative image of consistency (such as “not unpredictable” and “does not break his or her promises”). Appendix A shows an example of important search terms for each image. For additional information on the measurement of images of leadership in newspapers, the dictionaries, or the validation, see Aaldering and Vliegenthart (2016).

The occurrences of the leadership images are coded as a proportion of the total amount of references to the party leader, that is, the measurement of the images in the media relative to leader visibility. In total, we found 144,100 newspaper articles with a reference to at least one party leader in the studied period, of which 23,232 articles contained a total of 32,283 leadership images. Appendix B presents relevant descriptive statistics on how party leaders are discussed in newspapers in terms of their character traits.

### Panel Data

We connect this content analysis of media coverage of party leaders to the extensive 1VOP panel survey data on respondents’ vote intentions. The respondents in 1VOP enlisted via self-application and the 1VOP data set emerged from 110 waves. Since this article focuses on the effects of media coverage on electoral change, we selected respondents who participated in at least two waves and that read at least one of the national newspapers, leaving us with 53,698 respondents. While there is self-selection bias, this bias largely disappears once we control for earlier vote choice (cf. van der Meer et al. 2012). A challenging aspect of 1VOP for the purposes of our analysis is that it is unbalanced; respondents do not participate in all waves. To address this, we restructured the data into “personalized waves”; for each respondent, we included only those waves in which he or she actually participated.

### The Combined Media and Voter Data set

The media information was reshaped to be consistent with the respondents’ profiles. First, each respondent is connected only to the content of the newspapers that that respondent claims to read on a regularly basis. In other words, we calculated respondents’ personal media diet (cf. De Vreese and Boomgaarden 2006; Takens et al. 2013; Van Spanje et al. 2013). Unfortunately, it is unknown how many days a week respondents on average read the newspapers they indicated they read on a regularly basis, and thus, the media content cannot be weighed accordingly. Second, we adjusted the media information to align with the time periods of each respondent’s personalized waves by averaging the media information over the period between two personalized waves, weighted by the number of days. The main independent variables are thus party leaders’ media visibility and the ten mediated leadership images in the period prior to the 1VOP wave.^7^ Third, we stacked the data to the level of political parties so that the unit of analysis in our merged data set is a trinity of Respondent × Participated wave × Party. As a result, our main analyses are based on 11,691,944 cases. The dependent variable (the respondent’s intention to vote for the party) is coded as a dichotomous variable. Appendix C presents an example of the structure of the merged data set.

While the 1VOP data set provides unique opportunities for studying electoral dynamics, it is rather limited in the availability of independent variables. Ideological positions, issues, and party identification are not included in the data. Since party identification is not a very useful concept in the Dutch case (e.g., Thomassen 1976; Thomassen and Rosema 2008), we do not find this problematic. However, models without left–right distance and issue positions would normally be considered to be underspecified. We resolve this problem by including a lagged dependent variable in the model. The lagged dependent variable largely captures the effects of other predictors of the vote that are relatively stable over time, including socioeconomic demographics.^8^ In addition, by including the lagged dependent variable, we are effectively modeling change, which is our central interest in this article. Furthermore, we control for respondent-specific features: the total amount of waves of the 1VOP in which the respondents participated, membership in the specific party, voting for the specific party in the previous parliamentary elections, and various demographics (gender, age, and level of education).

### Analysis

This merged data set opens up the possibility to test the effects of mediated leadership images on shifts in vote intentions on the individual level. These types of questions are usually difficult to study due to the lack of sufficient statistical power. This data set does not suffer from this problem, with its sizable number of respondents, regular waves, high number of party leaders, and enormous amount of media information. However, a drawback of this data structure is that the number of days between two waves differs strongly between respondents and within respondents over time; (personalized) waves are not fixed in time but depend on the participation of the respondent in 1VOP.

In this research, we employ logistic regression analyses, which pose some methodological challenges. First, unobserved heterogeneity, that is, the influence of relevant variables that are not included in the model, is mainly addressed by (i) including the lagged dependent variable, (ii) including party fixed effects, and (iii) clustering the standard errors by respondents. Second, to test whether the unequal time periods between (personalized) waves affects the results, a robustness check with interaction effects between the media variables and the number of days between waves is performed. Another solution to the problem of unequal time periods is to include only the media data of a fixed number of days before the 1VOP wave or to use exponential decay on the media variables (see, for instance, Kleinnijenhuis and Walter 2014). However, the basic assumption underlying this strategy is that voters decide who to vote for or change party preference in the exact moment that they indicated in the survey. Since we know only whether voters changed their party preferences in the time between two waves but not when they did, we include the averaged media content of the whole period between waves.

## Results

Table 1 presents the results of the analyses. Model 1 includes the media visibility of party leaders and the general tone in which party leaders are described in terms of their character traits and the control variables. It shows first that the media visibility of party leaders has a small positive effect on vote intentions (even when controlling for previous party preference). Moreover, positive images of party leaders in newspaper reports have a significant positive effect on vote intention and, thus, increase the probability that a voter votes for the leader’s party, in line with H1. We expected to find asymmetrical effects for positive and negative images (H3), where negative images were assumed to exert a stronger effect than positive images. However, the results suggest otherwise. The negative effect of negative images in the media is smaller than the positive effect of positive images. Thus, positive descriptions of party leaders in terms of their leadership qualities are more influential than negative descriptions, contradicting our expectations but in line with the findings of Wattenberg (1991) and Aarts and Blais (2013), who also show stronger effects of subjective positive leader evaluations than of negative ones.

**Table 1.** Mediatized Party Leader Effects on Vote Intention.

Vote Intention for Parties	Model 1: General Tone	Model 2: Traits	Model 3: Campaign
	Log Odds (*SE*)	Log Odds (*SE*)	Log Odds (*SE*)
Vote for party (*t* − 1)	5.29***	5.30***	5.30***
	(0.01)	(0.01)	(0.01)
Media visibility party leader	0.01***	0.01***	0.01***
	(0.00)	(0.00)	(0.00)
Media visibility party leader			0.01***
× Campaign (1 = campaign)			(0.00)
General tone	0.39***		0.34***
Positive	(0.02)		(0.02)
General tone Positive			0.18***
× Campaign (1 = campaign)			(0.04)
General tone	−0.11***		−0.25***
Negative	(0.03)		(0.04)
General tone Negative			0.45***
× Campaign (1 = campaign)			(0.06)
Political craftsmanship		0.28***	
Positive		(0.03)	
Political craftsmanship		−0.34***	
Negative		(0.05)	
Vigorousness		0.62***	
Positive		(0.03)	
Vigorousness		−0.15***	
Negative		(0.03)	
Integrity		0.20***	
Positive		(0.04)	
Integrity		−0.13**	
Negative		(0.05)	
Communicative skills		0.29***	
Positive		(0.03)	
Communicative skills		−0.17**	
Negative		(0.06)	
Consistency		−0.60***	
Positive		(0.08)	
Consistency		0.64***	
Negative		(0.05)	
Membership party	1.34***	1.34***	1.30***
	(0.01)	(0.01)	(0.01)
Vote for party 2006	1.74***	1.74***	1.75***
(0 = *no*; 1 = *yes*)	(0.01)	(0.01)	(0.01)
Vote for party 2010	2.13***	2.13***	2.13***
(0 = *no*; 1 = *yes*)	(0.01)	(0.01)	(0.01)
Constant	−4.83***	−4.83***	−4.74***
	(0.05)	(0.05)	(0.05)
Pseudo Log-Likelihood	−977328	−976989	−947355
Number of observations	11,691,944	11,691,944	11,448,309
Number of respondents	53,698	53,698	53,203
Pseudo *R*^2^	.73	.73	.73

*Note.* The dependent variable is intention to vote for party (0 = *no*; 1 = *yes*). The standard errors are clustered by respondent; the models additionally control for the total amount of waves respondents participated in, the number of days in between waves, gender, age and level of education, and party fixed effects were added to the model (not shown here). The campaign period of the 2006 election campaign is excluded in model 3, as the corresponding routine time was not included in the content analysis.

* *p* ≤ .05. **p ≤ .01. ***p ≤ .001.

Another interesting feature of the model concerns the explained variance. The pseudo *r*-square indicates that approximately 73 percent of the variance in vote intention is explained with the model. This high percentage of explained variance is not caused by the media variables in the models but by the control variables, particularly the lagged dependent variable; a model that includes only the lagged vote intention explains 69.5 percent of the variance (not shown here). However, this does not mean that the effects of mediated leadership images do not matter.

**Figure 1.**
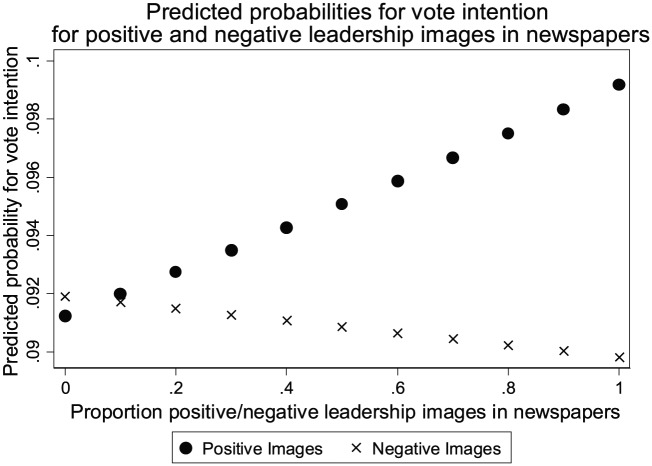
Predicted probabilities for vote intention.

To grasp their real-life impact, we plotted the predicted probabilities on vote intention for an average party in Figure 1. The figure shows that when party leaders are discussed positively in terms of their leadership qualities in all newspaper articles in which they are mentioned, the probability to vote for the leader’s party is on average approximately 1 percentage point higher than for the party whose leaders are not discussed positively in terms of leadership traits in any of the newspaper articles in which they appear (an increase in probability from .0912 to .0992). Although this observation may seem like a limited media effect, we must remember that we are analyzing the influence of the time between two waves of interviews in which the respondents participate. The average time lag between two waves is three months. Thus, in the unlikely event that a leader received only positive discussions of his or her character traits for a full year, the model predicts an increase in the electoral support for his or her party of 4 percentage points. The electoral effect of negative coverage in terms of leadership images in the media is estimated to be substantially smaller. The figure shows that the probability to vote for a leader’s party decreases from .0919 to .0898 when the proportion of the leader’s media visibility that also includes negative images increases from zero to 1.

Next, model 2 in Table 1 includes all ten distinct leadership images and shows significant positive effects of positive images on the vote intention for the leader’s party and negative effects of negative images on the leadership traits of political craftsmanship, vigorousness, integrity, and communicative skills, as predicted by H4. However, on trait consistency, we find opposing effects than hypothesized. Here, negative leader images increase the probability to vote for the leader’s party, while positive images decrease this likelihood, contradicting the basic notion of media influence; negative coverage results in a positive effect and vice versa. This effect remains consistent even when excluding parties one by one and when conducting an analysis with just this one trait rather than controlling for the others. We cannot provide an obvious explanation for this unexpected finding. The only explanation that we can think of is that the “inconsistency” of parties is a consequence of the need to find compromises to govern. In the Dutch multiparty system, which requires collaboration with political opponents, voters will usually understand that one has to compromise. The unwillingness to do so to remain consistent is perhaps not valued positively by voters.

To test the influence of campaign periods on the effects of mediated leadership images, we added interaction effects between the positive and negative mediated images and a dichotomous variable that measures whether it is a campaign period or a routine period (model 3 in Table 1). Campaign period is operationalized very broadly as the period in between the announcement of new elections (all cabinets in this time period ended prematurely) and Election Day. For this analysis, the short period before the 2006 election is excluded, as the corresponding routine period (2003-2006) was not included in the content analysis. The table shows that the effects of leadership images on vote intentions differ substantively in both periods. During times of routine politics, there is a positive effect of positive images and a negative effect of negative images, as predicted.

The results during campaign periods, on the other hand, tell a somewhat different story. The (positive) effects of positive images increases during campaign periods, supporting our expectation that mediated leader effects are stronger during election campaigns. However, while negative images have a negative effect during routine times, this effect is not augmented during campaign periods. Rather, the negative effect of negative images disappears during campaign periods.^9^ A possible reason could be that in campaign times, any evaluation of mediated leadership may be a cue for relevance (see also Kleinnijenhuis et al. 2007).

**Figure 2.**
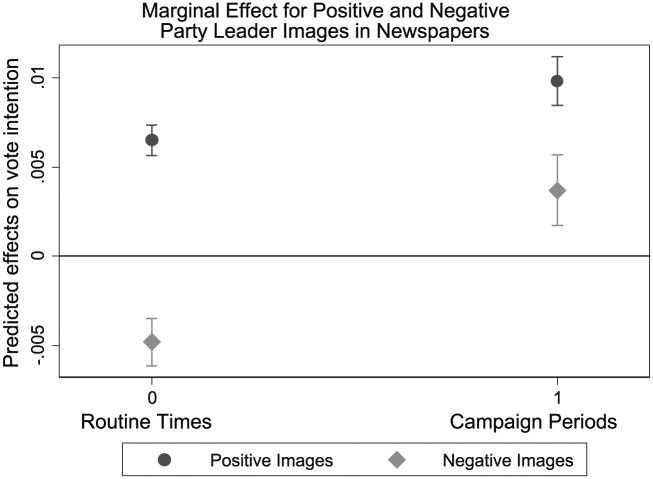
Party leader effects on vote intention during campaign periods and routine times. *Note.* The campaign period of the 2006 election campaign is excluded in this model, as the corresponding routine time was not included in the content analysis.

Figure 2 presents these effects graphically. The figure suggest that the effect of negative leadership images in the media would even turn positive during campaign times. However, while the unexpected positive interaction effect is robust against various model specifications, the marginal positive effect of negative news during campaign periods becomes insignificant in various robustness checks (see below). Thus, the evidence to conclude that politicians benefit from negative news during campaign periods is insufficiently robust, although the fact that negative news does not harm them during campaign periods is surprising in and of itself.

## Robustness Checks

We performed various robustness checks of our models. First, the results in the previous paragraph are based on the media coverage of all parties. However, not all parties are equally large and receive the same amount of media coverage. We checked whether the amount of media coverage influenced the results by adding interaction effects of general tone (positive and negative) with media visibility. The results confirm that media visibility impacts the effect sizes. To test whether the results are driven by an “overrepresentation” of small parties, we therefore performed additional analyses on a subset of exclusively large parties. The results are presented in Appendix D. Although the size of the coefficients differs somewhat from the coefficients in the main model, both the direction and the significance of all coefficients is robust. A first notable difference is the negative effect of negative images on communicative skills in model 2 (considerably stronger for large parties than for all parties). Second, the negative effect of negative images during routine times is stronger for large parties, while the positive effect of negative images in campaign periods is weaker. Appendix D depicts the effects graphically and shows that during campaign periods, negative leadership images have an insignificant effect. This finding suggests that the positive effect of negative leadership images in the media is driven mainly by smaller parties, in line with our earlier suggestion that in campaign times, any evaluation of mediated leadership may be a cue for relevance.

Second, to test whether the results are driven by specific parties, we jackknifed the analyses. Basically, we performed the analyses eleven times, excluding each party in turn. The results of the jackknifed analyses of model 1 are presented in Appendix E. The results show that the effects of media visibility and positive images in the media on vote intention are robust. All effects are positive, significant, and somewhat equal in size to the main effects presented in model 1 of Table 1. However, the negative effect of negative images in the media is robust for all jackknifed analyses, except for the analyses that exclude D66 and the VVD. In the former case, the effect becomes insignificant, while in the latter instance, the effect becomes positive, which would indicate that D66 and VVD are mainly responsible for the negative effect of negative images. We therefore performed a range of additional checks. In turn, jackknifed model 3, excluding D66 and VVD, suggests that these parties do not drive the negative effect of negative images during routine times but strongly impact the positive effect of negative images during campaign periods. Moreover, jackknifed model 2, excluding D66 and VVD, in turn strongly resembles our original findings with the exception that the negative effects of negative images on integrity and communicative skills become nonsignificant. However, overall, the jackknife procedure indicates that specific parties do not drive the results in our main analyses.

Third, the number of days between the two waves varies strongly between and within respondents over time in the 1VOP. To test whether these unequal time intervals affect the results, we performed additional checks by including interaction effects between (standardized) media variables and the (standardized) number of days between waves. Appendix F presents the results of this analysis. All (interaction) effects are very small but significant, indicating that the results are not independent from inequality in time intervals between the waves of 1VOP. The table shows, first, that when the number of days is at its mean (with a value of zero in the standardized variable), there is a positive effect of media visibility on vote intention. The interaction effect between media visibility and the number of days between waves is negative and significant, indicating that the positive effect of media visibility is strongest when the time period between waves is short, while the effect is weakest when this period is longer. Moreover, there is a positive effect of positive images and a negative effect of negative images when the number of days between waves is at its mean. When the number of days between waves increases, both effects are strengthened; thus, the positive effect of positive images becomes more positive, and the negative effect of negative images becomes more negative. However, these effect sizes are very small, and thus, the dependency on the number of days in between waves should not be overstated.

Fourth, one of the challenges of this data structure that the main analyses do not consider is the dependency of observations on the dependent variable. We stacked the data on political parties, which increased our data set eleven times. However, if a respondent indicated a vote intention for one party in a specific wave, this automatically implies that he or she would not vote for the other ten parties. To address this dependency, we test the robustness of our findings on models in which all media variables are centered around the mean per respondent/wave. Now, all variation between respondents and over time is accounted for, and we explain only the variance between parties within respondents and waves. Appendix G presents the results of these analyses and shows that leadership visibility and positive coverage increase the likelihood of voting for the leader’s party, while negative leadership coverage decreases this likelihood (model 1). In addition, model 2 of Appendix G is a replica of model 2 in Table 1 in terms of direction and significance. Model 3 and the figure in Appendix G show that the effects of negative images during campaign periods are similar to the effects in the main analyses.

Fifth, we also performed the analyses including respondent/party fixed effects in the model. Thereby, we exclude all variation between respondents and between parties and only model the variation within respondents over time for each party. Consequently, all respondent–party combinations without any change over time (i.e., when a respondent always votes for the party or when the respondent never votes for the party) are excluded, and the analyses are performed on a substantially smaller subset of observations. Appendix H presents the results. Although there are slight changes in the coefficients when respondent/party fixed effects are added to the models, the direction and significance of all media effects in models 1 to 3 remain equal to the results in Table 1.

Finally, we test whether including party leader fixed effects (instead of party fixed effects) changes the results. These models will no longer capture the effects of changes in the leadership of a party and electoral consequences that are due to a different leader with different media coverage. Appendix I presents the results of the analyses with leader fixed effects. Model 1 shows that positive images result in increased party support, as in the main results. It also shows that the effect of negative images in the media is insignificant, contrasting previous findings. However, when we inspect the effects of negative images in campaign and routine times (model 3), the findings are largely similar to the main results: during times of routine politics, there is a negative effect of negative leadership images in the media, while these effects become positive during campaign periods. In addition, model 2 of Appendix I presents very similar results as model 2 in Table 1 in terms of direction and significance.

Overall, we conclude that the effects in the main analyses are generally robust to alternative case selections and model specifications, with one important exception. The positive effect of negative images of leadership during campaign periods is not robust to all alternative model specifications. Particularly, it becomes insignificant when only large parties are included. Thus, although we conclude that the negative effect of negative images disappears during election campaigns, there is insufficient evidence to conclude that the effect becomes positive during campaign periods.

## Conclusion

Mediated leadership images affect citizens’ vote intentions. The success of party leaders is inherently tied to the mediated environment, in which media are voters’ primary source of political information. Generally, positive leadership images stimulate support for that leader’s party, whereas negative images undermine support. Thus, these newspapers do not only influence what their readers think about but also how they think about it.

However, the media’s influence is not unconditional. During campaign periods, negative leadership images no longer have a negative effect on subsequent vote intentions. We can only speculate about the reasons why the negative effect of negative leadership images is dampened during an election campaign. It may be that the mediated discussion of the leadership traits of a party leader may signal a cue for political relevance during campaign periods, indicating that the party leader is important enough to elaborate upon, even when negative images are put forward. Most of these negative leadership images may even be initiated by political opponents during campaign periods via negative campaigning (e.g., Lau et al. 2007), while during routine times, these evaluations are made mainly by “objective” journalists. Alternatively, other, more strategic, considerations might come into play during campaign periods, as a result of which the “regular” mechanisms by which leadership images affect voters no longer apply.

We were able to isolate these mediated leader effects by combining an extensive computer-assisted content analysis of Dutch newspapers in the 2006 to 2012 period to measure leadership images with the large-scale 1VOP panel data set that measures respondents’ newspaper readership and their vote intentions in 110 waves. As a result of its large scope of media data, its many respondents, its many survey waves, and the large number of competing party leaders, the merged data set provided us with the statistical power needed to isolate mediated leader effects on the individual level.

The contribution of this research is twofold. First, this study extends our knowledge of the effects of party leaders on the electoral support for their party. To date, the literature has predominantly studied the tail end of this effect, that is, the effect of respondents’ subjective leadership evaluations on their vote intentions. We show that mediated leadership images have a considerable influence on these vote intentions as well. By focusing on mediated (rather than subjective) leadership images in a longitudinal model, we can be more certain about the causal direction of this effect. The next step in the literature should be an in-depth study of the causal mechanism, that is, the extent to which media coverage affects voters’ perceptions of party leaders.

Second, there is a more general scholarly debate on the extent to which media content influences media users. This article shows not only that affective news coverage has a direct effect on voting behavior but also that these effects depend on the wider context, that is, the presence of an election campaign. This indicates that the timing of media effects in the broader socio-political context is a factor to consider. Particularly to studies of media effects on electoral behavior, more research is needed to understand how media affects political behavior not only during campaign periods but also in times of routine politics.

We have argued that the Dutch case has several features that are likely to dampen mediated leader effects and other features that could boost those effects. In combination, we do not expect these effects to be particularly strong, nor particularly weak, compared with other contexts. Yet, this can only be scrutinized by replicating our study in different contexts.

Besides the features of the Dutch context, we also have to consider the fact that our estimated effect sizes are based on respondents’ personal media diets. Since voters’ choice of newspapers is likely to be related to their political preferences, the reinforcement effects of party leaders will probably be larger than their defection effects. Political leanings inherent in news coverage probably result in strengthening party preferences rather than changing them. This could make our estimates somewhat conservative.

Another reason to consider our estimates conservative is our explicit limitation of media content to newspaper articles, thereby excluding televised or digital content. It would be interesting to examine how mediated leadership images in other media outlets affect voters, especially televised images of leadership, as television is the voters’ principal political information source and is less connected to political parties than newspapers are (Gunther and Mughan 2000).

While this article shows that mediated leadership images affect vote intentions, it cannot distinguish between pure media effects and pure leader effects. In other words, we are agnostic as to whether these mediated leadership images are neutral reflections of party leaders or are largely constructed in media reports instead. To disentangle the influence of party leaders themselves from the influence of journalists who report on them, future studies should compare mainstream media content with media content controlled mainly by parties. In addition, the content analysis does not distinguish the positive and negative coverage of the character traits of party leaders from the more general positive and negative coverage of parties and their leaders. When the tone in the reporting of the character traits of party leaders coincides strongly with the tone in which parties and their leaders are described more generally, excluding the latter from the analysis might affect the findings. A content analysis that is even more extensive than the one applied in this article, which includes both party coverage and leader coverage, could unravel these distinct influences on the vote decision.

We know that mediated leadership images matter; the next step is to tease out the conditions in which they matter. When are voters most likely to be swayed by mediated leadership images? There are at least three relevant levels on which this question must be studied: the macro-level, comparing mediated leader effects in different political systems and media systems; the meso-level, comparing mediated leader effects of male and female party leaders and testing the influence of trait ownership (Hayes 2005); and the micro-level, comparing mediated leader effects between, for instance, voters with high and low political sophistication or partisans and nonpartisans.

## Notes

1. Party leader effects are defined as the “added value, in electoral terms, that a specific (. . .) candidate is able to bring his or her party or coalition through the effectiveness of his or her public image as appraised at that specific time” (Barisione 2009: 474).

2. Moreover, this affective dimension of media influence is stronger than the substantive dimension, which implies that it is not media visibility that matters most but rather the tone of the coverage of party leaders in media reports (McCombs et al. 1997).

3. Volkskrant, Telegraaf, NRC Handelsblad, NRC Next, Algemeen Dagblad, Trouw, Parool, Financieele Dagblad, Spits, Metro, Pers, and DAG.

4. *Trots op Nederland* (TON), the party of former VVD politician Rita Verdonk, was also included even though this party did not earn an elected seat in parliament in the National Parliamentary elections of 2010. TON and Verdonk were included because Verdonk, as an independent Member of Parliament after she was excluded from the VVD in 2007, was very visible in the media and an influential politician for some time, while her party received up to about 17 percent of the votes in the polls in 2008.

5. Party leaders are conceptualized as chairmen of the party in Parliament (for opposition parties during routine times); chairmen of the party in Parliament or a (vice-) prime minister (for coalition parties during routine times); and party leaders during campaign periods.

6. The dictionaries measuring the leadership images are available on request.

7. Bear in mind that we cannot distinguish between pure leader effects and pure media effects. This article is agnostic regarding whether or not the media primarily portray leaders conform reality or not.

8. However, unfortunately, the model does not allow us to control for other short-term influences on the vote, such as the party’s popularity in the polls.

9. We checked whether this surprising result was driven by a specific election campaign, but it was found in the campaign periods in both 2010 and 2012.
